# Phenolic and Antioxidant Analysis of Olive Leaves Extracts (*Olea europaea* L.) Obtained by High Voltage Electrical Discharges (HVED)

**DOI:** 10.3390/foods8070248

**Published:** 2019-07-08

**Authors:** Irena Žuntar, Predrag Putnik, Danijela Bursać Kovačević, Marinela Nutrizio, Filip Šupljika, Andreja Poljanec, Igor Dubrović, Francisco J. Barba, Anet Režek Jambrak

**Affiliations:** 1 Faculty of Pharmacy and Biochemistry, University of Zagreb, 10000 Zagreb, Croatia; 2 Faculty of Food Technology and Biotechnology, University of Zagreb, 10000 Zagreb, Croatia; 3 Teaching Institute for Public health of Primorje-Gorski Kotar County, 51000 Rijeka, Croatia; 4 Nutrition and Food Science Area, Preventive Medicine and Public Health, Food Sciences, Toxicology and Forensic Medicine Department, Faculty of Pharmacy, Universitat de València, Avda. Vicent Andrés Estellés, s/n, Burjassot, 46100 València, Spain

**Keywords:** high voltage electrical discharge, olive leaves extracts, green solvents, eco-extraction, sustainability

## Abstract

Background: The aim of this study was to evaluate high voltage electrical discharges (HVED) as a green technology, in order to establish the effectiveness of phenolic extraction from olive leaves against conventional extraction (CE). HVED parameters included different green solvents (water, ethanol), treatment times (3 and 9 min), gases (nitrogen, argon), and voltages (15, 20, 25 kV). Methods: Phenolic compounds were characterized by ultra-performance liquid chromatography-tandem mass spectrometer (UPLC-MS/MS), while antioxidant potency (total phenolic content and antioxidant capacity) were monitored spectrophotometrically. Data for Near infrared spectroscopy (NIR) spectroscopy, colorimetry, zeta potential, particle size, and conductivity were also reported. Results: The highest yield of phenolic compounds was obtained for the sample treated with argon/9 min/20 kV/50% (3.2 times higher as compared to CE). Obtained results suggested the usage of HVED technology in simultaneous extraction and nanoformulation, and production of stable emulsion systems. Antioxidant capacity (AOC) of obtained extracts showed no significant difference upon the HVED treatment. Conclusions: Ethanol with HVED destroys the linkage between phenolic compounds and components of the plant material to which they are bound. All extracts were compliant with legal requirements regarding content of contaminants, pesticide residues and toxic metals. In conclusion, HVED presents an excellent potential for phenolic compounds extraction for further use in functional food manufacturing.

## 1. Introduction

Plant extracts obtained from fruits, leaves, flowers, woods, roots, resins or seeds of known medicinal properties are responsible for many health benefits, like reduction of hypertension, prevention of cardiovascular disease, suppression of different types of cancer and viral disease, etc. Classical/conventional extraction (CE) methods are not only time and energy demanding, but are also prone to usage of solvents, resulting with overall hazardous/toxic effects on the environment. Therefore, there is a growing interest to utilize plant extracts with “green chemistry“ in science-based production of functional foods, food supplements, cosmetics, perfumes, nutraceuticals, and pharmaceuticals in a sustainable ways [1,2,3,4,5,6,7].

Olive tree (*Olea europaea* L.) is cultivated from antiquity in many parts of the world, but Mediterranean region is the main agricultural area for its production. Mediterranean region is also known for its diet with olives containing nutritional, health and antioxidant benefits, while being rich in with phenols naturally originated from olive plant [8,9,10]. Olive leaves (OLs) which may be considered as both, waste from olive oil production and medicinal and aromatic herbs, contain a large variety of phenols including oleuropeosides (oleuropein and verbascoside); flavonols (rutin); flavones (luteolin-7-glucoside, apigenin-7-glucoside, diosmetin-7-glucoside, luteolin and diosmetin); flavan-3-ols (catechin), substituted phenols (tyrosol, hydroxytyrosol, vanillin, vanilic acid and caffeic acid), oleoside and secoiridoid glycoside (oleuricine A and oleuricine B) [1,2,9,10,11,12,13].

It is well known that polyphenols as antioxidants may prevent or minimize oxidative damage/stress at cellular levels, resulted from generated concentration imbalance between reactive oxygen (ROS) and nitrogen (RNS) species and cell antioxidants. Numerous phenolics in OLs have strong radical scavenging activity, showing that olive phenolics exhibit more beneficial effects in form of a mixture (e.g., olive leaves extract; OLE) than isolated as a single phenolic compound. Olive leaves extract have a synergistic capacity in the elimination of free radicals, that is more superior to the antioxidant capacity of the vitamin C and E [3,7,8,9,10,11,12]. Consequently, OLE as an antioxidant may reduce the risks for harmful health effects [3,10,11]. Also, recent studies on OLE confirmed potential coadjuvant use for cervical cancer treatments [12], inhibitory effects on the obesity [13], suppression of inflammatory cytokine production and the activation of NLRP3 inflammasomes in human placenta [14], potential as preventive therapy for neurodegenerative diseases [15], protective quality of testis and sperms [16], protection against intoxication of liver by carbon tetrachloride, kidney by cadmium, and brain by lead poisoning [17,18,19]. 

Furthermore, OLE polyphenols are useful as natural food additives that have antimicrobial and antioxidant properties. For instance, OLE are good for fermentation and oxidative processes of table olives and for improving their nutritional properties [20], i.e., as replacement of synthetic additives and the shelf life extension of fish patties [21] and salmon burgers [22]. OLs as by-products/waste generated during olive oil production contains even higher amounts of polyphenols than appreciated olive oil. Therefore, there is a great interest for their extraction from OLs and especially with application of eco-friendly and sustainable processes [2,23,24,25]. 

However, green chemistry only applies in a part of the life cycle of a single natural product [4] which also includes food safety aspects. Therefore, OLEs must be safe with respect to contaminants, pesticide residues and toxic metals (Pb, Hg, Cd, etc) [26,27]. Nonetheless, it is important to note that OLs are a rich source of various minerals (Ca, K, Na, P, S, Cl, Mg, Al, Si, K, Mn, Fe) and trace elements (Ni, Cu, Sr, Ba, Cr, Zn, Rb, Th, Co, Ng, Cs, La, Ce, V, Nd) [28].

In a context of the Chemat’s principles of green chemistry, an extraction is defined as:

“*Green extraction is based on the discovery and design of extraction processes which will reduce energy consumption, allows use of alternative solvents and renewable natural products, and ensure a safe and high-quality extract/product*”

[4].

Current regulations including REACH (Registration, Evaluation, Authorization and Restriction of Chemicals, CLP (Classification, Labelling and Packaging of substances and mixtures), IPPC (Integrated Pollution Prevention and Control) [29], and notation of BAT (Best Available Technology) have direct progressive impact in diminishing the consumption of organic solvents and Volatile Organic Compounds (VOC) [4]. Hence, they aim to save human health and environment, both in research and development (R&D), as well as in production. Among green solvents, the agro- or bio-solvents play an important role for the replacement of organic solvents. Ethanol is a well-known, common bio-solvent, classified as environmentally preferable. It is obtained by the fermentation of sugar-rich materials such as sugar beet and cereals. Although it is flammable and potentially explosive, ethanol is used on a large scale due to synthesis in high purity, low price, and complete biodegradability [4].

In order to improve CE, modern non-conventional extractions were developed to reduce the mass transfer limitations and to increase yields in shorter time with minimal consumption of solvents. These advanced techniques include ultrasound-assisted extraction (UAE), microwave-assisted extraction (MAE), sub- and super-critical fluid extraction (SFE), pressurized liquid extraction (PLE), pulsed electric fields (PEF), and high voltage electrical discharges (HVED) [1,30,31]. Although there are limitations of innovative technologies for industrial implementations (e.g., high costs, control of process variables, lack of regulatory approval, and consumer acceptance) [32], HVED (cold plasma) is promising green technique for the extraction of biologically active compounds (BACs) with green solvents. Thus, it has the potential for development of green chemical engineering and sustainable production [25]. PLE, in example, would yield the greatest possible amounts of flavonoids (25.66 mg/g) in one extraction cycle, that lasts for 15 min at 100 °C. If extraction, for PLE, would be-taken to obtain the most of flavonols (9.22 mg/g), that can be achieved with one extraction cycle, that lasts for 5 min at 87 °C [23].

Developments of non-thermal technologies, such PEF and HVED, have been advancements for industry and academia in meeting the challenges of producing safe and high-quality food [1,2]. HVED is an emerging technology and broad designation for pulsed mode plasma systems, but it is mainly known as “corona discharge” due to its discharge reminiscent of crown that surrounds the cathode wire with pulsed DC power supply [33].

It is well known that HVED extraction is one of the applications of the liquid phase discharge technology where a phenomenon of electrohydraulic discharge might occur in the water by the effect of high voltage pulsed electric field which is accompanied by several secondary phenomena. Additionally, UV light can lead cell inactivation by damaging to the DNA, shock waves and strong liquid turbulence can cause products fragmentation as well as mechanical destruction of cell tissues, and the high density of radicals can damage to the cell by cell oxidation. These are reasons why HVED may destruct cellular structure and enhance mass transfer from the cell to the solution, thus greatly improving the yields of BACs [34]. Pioneers of HVED extraction summarized many aspects of HVED extraction, but recent review puts emphasis on introducing different extraction devices, including batch, continuous and circulating extraction systems, generalizing the critical processes factors and recent applications, discussing the advantages and disadvantages of HVED extraction as well as the future trends of HVED assisted extraction technique. Although HVED is very potent extraction method with so many advantages, the authors also discussed some disadvantages and problems needed to be solved for its future development that will benefit various fields such as food and medical industries [34].

Therefore, the aim of this study is to evaluate HVED in the extraction efficiency of phenolic components from autochthonous Mediterranean OLs by varying: (i) concentrations of ethanol; (ii) voltages applied; and (iii) treatment times. Phenolic content in OLE was characterized by ultra-performance liquid chromatography-tandem mass spectrometer (UPLC-MS/MS), while antioxidant tests were conducted to analyze the antioxidant potency. Near infrared spectroscopy (NIR) was used for the prediction of an extract quality and analyzed by principal component analysis (PCA) and all results were controlled against CE as standard.

## 2. Materials and Methods 

### 2.1. Plant Material

Dried olive leaves (*Olea europaea* L.) were provided locally from Mediterranean area (Zadar county, Croatia). Herbs were stored in polyethylene bags in a dark and dry place until extractions. Herbs were milled using knife mill (Grindomix GM 300 – RETSCH; Retsch GmbH, Haan, Germany) before HVED treatment. Plant particle size distribution was: *d*(0.9) ≤ 330.563 μm; *d*(0.5) ≤ 118.540 μm; *d*(0.1) ≤ 23.105 μm measured by the laser particle size analyzer (Malvern, Mastersizer 2000, Germany). Prior to HVED extraction, leaves were weighted (1 g) and mixed with 50 mL of extracting solvent, as distilled water, 25% and 50% aqueous ethanol (*v*/*v*) at room temperature (22 °C).

### 2.2. Conventional and HVED Assisted Extraction

The CE was used as a control procedure to compare the efficiency of HVED under the same conditions with respect to solvent type and treatment time. Therefore, the CE was conducted by varying: (i) solvent type (0, 25 and 50% of ethanol (*v*/*v*)); (ii) stirring time (3 and 9 min) at room temperature (22 °C). Magnetic stirring was used to provide effective stirring of herb and extraction solvent during CE. HVED assisted extraction (plasma) generator “IMP-SSPG-1200” (Impel group, Zagreb, Croatia) was used for rectangular pulses from direct current (DC) as high voltage (HV) generator. Maximum adjustable current was 30 mA up to voltage of 25 kV. Frequency was 100 Hz; pulse width was 400 μs; voltage was 15 kV and 20 kV for argon gas; and 20 kV and 25 kV for nitrogen. 

**Figure 1:**
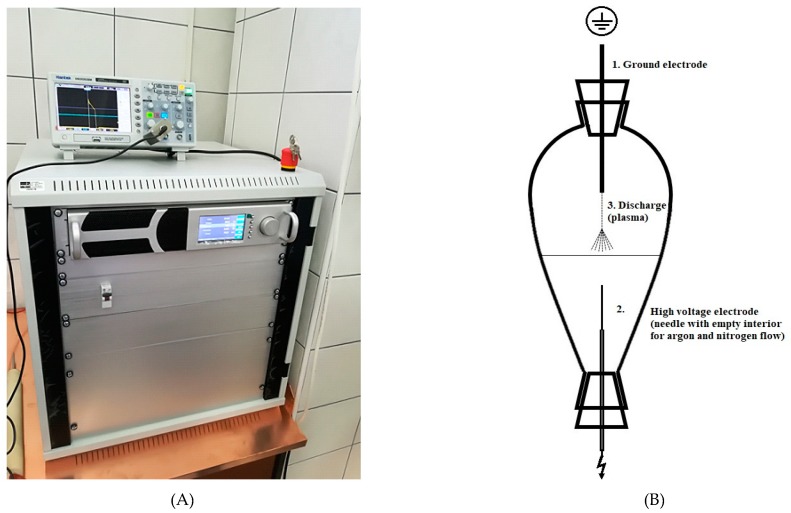
Set-up of generator and reactor for high voltage electrical discharges (HVED) treatments. (**A**) HVED and plasma generator “IMP-SSPG-1200” (Impel group, Zagreb, Croatia); (**B**) Beaker shaped reactor.

Parameters were chosen based on conducted preliminary experiments with different HVED parameters (frequency, voltage, pulse length, distance between electrodes), as well as mass to solvent ratio. From more than 100 experiments, mentioned parameters were chosen. Regarding gases: it is important to have significant voltage to obtain discharge, if too low, there will be no discharge. For that reason, 15 kV and 20 kV were chosen for argon, and 20 kV and 25 kV for nitrogen. For ethanol concentration, pharmacopeia regulations were followed. The mixture of leaves and solvent was transferred to beaker shaped reactor V = 100 mL. This reactor was opened on both sides and fitted with silicone tops (diameter 1 cm). They were used for easier mounting of the electrode from the top and needle form the bottom. Both, argon or nitrogen were introduced through the needle with a flow of 5 L min^−1^. The gap between electrodes was 15 mm, while the set-up of generator and reactor is shown in Figure 1. The high voltage probe (Tektronix P6015A) connected to the oscilloscope (Hantek DS05202BM) was used to measure the output voltage (data not shown). Physical properties as pH, conductivity (μs/cm), temperature and power consumption of the instrument were monitored before and after HVED treatment and modified CE.

#### Experimental Design and Statistical Analysis

The experiment was designed in STATGRAPHICS Centurion (StatPoint Technologies, Inc, Warrenton, VA, USA) software. Multifactorial design consisting of 12 experimental trials using per each gas (argon and nitrogen). The three chosen independent variables were: (i) concentration of ethanol (0, 25 or 50%), voltage applied (15 kV or 20 kV for argon, and 20 kV or 25 kV for nitrogen) and treatment time (3 and 9 min) (Table 1). Both extractions, HVED and CE were performed in duplicates. The model was fitted by multiple linear regressions (MLR). Calculations were done at 95% of confidence level. 

Statistical analysis was done in STATGRAPHICS Centurion software (StatPoint Technologies, Inc, Warrenton, VA, USA). 

### 2.3. Determination of Total Phenolic Content (TPC)

Total Phenolic Content (TPC) of OLE was determined using Folin-Ciocalteu method (FC) as previously described [35] with slight modification. Briefly, a volume of 0.1 mL of extract (appropriately diluted) was mixed with 0.2 mL of FC reagent. After 3 minutes 1 mL of 20% Na_2_CO_3_ (*m*/*v*) was added. After thorough mixing by vortex, the reaction mixtures were incubated at 50 °C for 25 min, followed by absorbance reading at 765 nm against blank. The blank contained 0.1 mL of extraction solvent instead of an extract. The calibration curve was prepared by gallic acid standard solutions (50–500 mg/L) solubilized in ethanol. The absorbance was measured for each standard solution following the same procedure as for extracts. The concentration of TPC was expressed in mg of gallic acid equivalents per g of sample (mg GAE/g of sample).

### 2.4. Determination of Antioxidant Capacity (AOC)

#### 2.4.1. DPPH (2,2-Diphenyl-2-Picrylhydrazyl) Free Radical Assay

DPPH assay of OLEs was determined according to the previously reported procedure [36]. An aliquot (0.75 mL) of OLEs (appropriately diluted) was mixed with 1.5 mL of 0.5 mM DPPH methanolic solution. After mixing, the solutions were stored in the dark for 20 min at room temperature and then, the absorbance was measured at 517 nm against 100% methanol as a blank. The methanol solution of Trolox (25–200 µM) was used for the calibration curve. The absorbance values for the extracts were subtracted from the control sample (0.75 mL of 100% methanol and 1.5 mL of 0.5 mM DPPH). The results were calculated using the calibration curve for Trolox and expressed as µmol of Trolox equivalents per gram of samples (µmol TE/g of sample). 

#### 2.4.2. Ferric Reducing Antioxidant Power (FRAP) Assay

The FRAP assay was conducted according to the literature [37]. Prior to analysis, the FRAP reagent was prepared by mixing 0.3 M acetate buffer (pH = 3.6), 10 mM 2,4,6-tri(2-pyridyl)-1,3,5-triazine (TPTZ) solution in 40 mM hydrochloric acid and 20 mM FeCl_3_ in ratio 10:1:1. Furthermore, the FRAP reagent (2080 µL) was mixed with 240 μL of distilled water and 80 μL of appropriately diluted OLE. The mixture was vortexed and allowed to stand for 5 min at 37 °C prior to absorbance measurement at 595 nm. The amount of extract was substituted by the same amount of extraction solvent in blank. The calibration curve was made by preparing a standard aqueous solution of FeSO_4_·7H_2_O (25–750 µM) where absorbance was measured following the same procedure as described for extracts. FRAP values were calculated according to the calibration curve for FeSO_4_·7H_2_O and expressed as µmol of Fe^2+^ equivalents (FE) per g of sample (µmol FE/g of sample).

### 2.5. NIR

NIR spectroscopy were conducted using the Control Development Inc. (South Bend, IN, USA), NIR-128-1.7-USB/6.25/50 μm to record extract spectra using the SPEC 32 Control Development software. NIR spectra was recorded in the wavelength range of 904 nm to 1699 nm. Each sample was recorded in triplicate and afterwards was calculated the average spectrum which was used for further processing. Since NIR spectra provide a large amount of data of one sample, PCA analysis was used to recognize and extract the most important information from the measurements, thus reducing the amount of data [38]. The PCA analysis was carried out on a part of spectrum that shows the difference between the samples (absorbance from 1350 to 1699 nm). This method was implemented in XLStat (MS Excel 2010, Microsoft, Redmond, WA, USA; XLStat by Addinsoft, Paris, France).

### 2.6. UPLC-MS/MS

Determination of phenolic compounds was carried out on UPLC-MS/MS system Eskigent Expert Ultra LC 110 and SCIEX 4500 QTRAP. Separation of the phenolic fraction of olive leaves were performed by a Luna Omega 3μm Polar C18 100Å, 100 × 4.6 mm (column), thermostat column temperature 40 °C, automatic sampling temperature 4 °C, and injection volume 10 μL. Mobile phases consisted of: A 100% H2O with 0.1% HCOOH (*v*/*v*) and B 100% ACN with 0.1% HCOOH (*v*/*v*) with mobile phase flow 0.40 mL/min. Gradient was set as follows: 1 min 10% B, 2 min 10% B, 15 min 90% B, 25 min 90% B, 27 min 10% B, 30 min 10% B. Determination conditions for MS/MS detector were: negative atmospheric pressure ionization mode (API); ionization temperature: 500 °C, Ion Spray voltage: −4500V, drying gas temperature 190 °C and drying gas flow 9.0 L/min. Determination of phenolic compounds content in extracts was carried out once. Quantitation of phenolic compounds was processed using Multiquant 3.6 (SCIEX, Darmstadt, Germany) software. Partial validation of method was carried out by measuring 6 replicas of added (spiked) standard (10 ng/mL) of oleuropein and oleanolic acid in two different extracts with no trace of these compounds. Average recovery was 92.3% for oleuropein, 91.1% for oleanolic acid, standard deviation 1.108 ng/mL for oleuropein, standard deviation 0.956 ng/mL for oleanolic acid, RSD 1.20% for oleuropein and RSD 1.05% for oleanolic acid.

### 2.7. Colorimetric Evaluation of OLEs

Color parameters for all trials (pure extract) was measured by Konica Minolta colorimeter (Model CM 3500d, Konica Minolta, Tokyo, Japan) at CIE Standard Illuminant D65 by 8 mm thick plate. All measurements were conducted in the Specular Component Included (SCI) mode as previously reported [39]. Colorimetric values (L*, a*, b*) were measured and color change after HVED treatment (∆C, ∆H and ∆E) against untreated extracts (CE) was calculated. ∆L, ∆a and ∆b presents the difference between colorimetric value of HVED extracts and untreated (CE) extracts. Hue (H) was calculated from:

(1) $$H=\arctan\;\left(\frac{b^{*}}{a^{*}}\right)$$

Chroma (C) and the differences in tone color (∆C) are calculated based on the following formula:

(2) $$C=\sqrt{a^{2}+b^{2}}$$

∆C = C_HVED extract_ − C_untreated extract_ (3)

The total color difference (ΔE) was calculated on the basis of the measured parameters:

(4) $$\Delta E=\;\sqrt{\Delta L^{2}+\Delta a^{2}+\Delta b^{2}}$$

Saturation (ΔH) was determined based on the formula below:

(5) $$\Delta H=\;\sqrt{\Delta E^{2}-\Delta L^{2}-\Delta C^{2}}$$

### 2.8. Electrophoretic Light Scattering (ELS)

ELS measures the electrophoretic mobility of particles in dispersion or molecules in solution in the way that combines light scattering with electrophoresis. The OLE is introduced into the cell containing two electrodes. When an electric field is applied across the electrodes every charged particle or molecule will migrate towards its oppositely charged electrode with velocity which is dependent upon its charge. The measured electrophoretic mobility is converted to zeta potential using established theories. Zeta-potential measurements of all extracts were made on a Malvern Zetasizer Ultra (Malvern Panalytical, Malvern, UK) in a disposable folded capillary cells, thermostatted to 25 °C, at the forward angle (13°).

### 2.9. Dynamic Light Scattering (DLS)

DLS is a non-invasive analytical technique for measuring the size of particles and molecules in suspension which undergo Brownian motion (random movement of particles). It requires accurate and stable temperature because of sample viscosity. The velocity of the Brownian motion is defined through a property known as the translational diffusion coefficient (D). The size of a particle (hydrodynamic diameter) is calculated from the translational diffusion coefficient by using the Stokes-Einstein equation. Measurements of all extracts were made on a Malvern Zetasizer Ultra from Malvern Panalytical, UK in a disposable folded capillary cells, thermostatted to 25 °C, at the non-invasive back scatter (NIBS, 173°).

### 2.10. Determination of Pesticides and Metals in OLs 

The content of the pesticides was measured by modified procedures with following national regulations HRN EN ISO 12393-1,12393-2 and 12393-3: 2013, i.e., extraction with petroleum ether/dichloromethane and determination using the GC-ECD Varian CP-3800 instrument. Metal trace content was determined according to the HRN EN ISO 14084: 2005 procedure, or by wet sample digestion by HNO_3_ (microwave digestion) with Microwave reaction system Anton Paar, Multiwave 3000. Determination of metals were conducted on the Perkin Elmer AAS Analyst 800 and ICP-MS Perkin Elmer NexION 300X (PerkinElmer, Inc. 940, Waltham, MA, USA), while Hg traces were determined by the Leco AMA 254 Hg analyzer (LECO, St. Joseph, MI, USA).

## 3. Results and Discussion

### 3.1. Influence of HVED Treatment on Physical Parameters of OLEs

HVED was applied as an emerging non-thermal technology, with the aim to reduce extraction time and enhance extraction efficiency at lower temperatures than it is usual for CE. Data for pH, electrical conductivity, temperature, and power are given in Table 2. 

### 3.2. Influence of HVED Treatment on TPC and Antioxidant Activity of OLEs

The influence of individual process parameters (treatment time, applied voltage, ethanol content) and their interaction were evaluated for significance (*p* ≤ 0.05). It is important to emphasize that during 3 and 9 min of treatment time; temperature did not exceed 30 °C. In Table 3, results for extraction yield, TPC and antioxidant activity (DPPH and FRAP) for CE and HVED extracts were given. It can be seen that by using 50% ethanol (*v*/*v*) and HVED with argon for 9 min, high extraction yields were achieved as compared to CE-extracts. Also, 20 kV treatment for 3 min, with nitrogen gas gave 2,5-fold higher yield in comparison to CE extracts. A recent study confirmed HVED as an extraction technique with significant increase in yield due to disruptions of sample cells under electrical breakdown and enhanced mass transfer [40].

All HVED treated samples resulted with higher TPC values than CE samples which implies that HVED could be considered as an effective extraction tool at room temperature. The phenomena behind HVED is electroporation, where strength of electric field is directly proportional to the poration of plant cell membrane [1]. Moreover, it was found that higher ethanol/water ratio promote better polyphenolic extraction, thus gave extracts with higher TPC content. Similar results were observed by Garcia-Castello et al., who found that higher ethanol concentration favored flavonoids’ extraction from grapefruit (*Citrus paradisi* L.) solid wastes [41]. Authors explained that reduced dielectric constant of the solvent caused by ethanol could led to enhanced solubility and diffusion of polyphenols from plant matrix. With respect to pure water as an extraction solvent, it is clear that 2-6 times higher TPC values were determined for HVED extraction systems (OLN2, 3, 4 and 10; OLA2, 3, 4 and 10) in comparison to CE samples.

### 3.3. Influence of HVED Treatment on Soluble Phenolic Compounds in OLEs Analyzed by UPLC-MS/MS 

A UPLC-MS/MS analysis of individual phenolic compounds has shown that main constituents of phenolics in OLEs were as follows: apigenin, diosmetin, hydroxytyrosol, luteolin, oleanolic acid, oleuropein, and quercentin (Table 4). It is obvious that there is an increase in content of phenolics with increase in ethanol concentration, likely due to increased solubility of phenolic compounds [42]. Oleuropein is the main constituent of OLE. Similar trend is shown for HVED treatment, with 50% ethanol (*v*/*v*), where higher concentrations of hydroxytyrosol and oleuropein were observed Hydroxytyrosol, which is a precursor of oleuropein, is scavenger of superoxide anions, and inhibitor of neutrophils and hypochlorous acid-derived radicals [29]. It is also important to emphasize that the highest values of phenolics in OLEs for HVED treated samples, were shown for OLA8 (argon gas, 9 min treatment, 20 kV, 50% ethanol) sample and OLN1 (nitrogen gas, 3 min, 20 kV, 50% ethanol). HVED extraction at the highest voltage (25 kV) and 9 min extraction time, resulted in degradation of oleanolic acid in OLE. This HVED technology with pulsed rapid discharge voltages (from 20 to 80 kV/cm electric field intensity) is based on the phenomenon of electrical breakdown in liquids which induces physical and chemical processes that affect both the cell walls and the membranes, while freeing intracellular components [43]. Moreover, HVED generates hot and localized plasma during photonic dissociation of water, with emission of the UV light and -OH radicals. At the same time, HVED will create shockwaves and pyrolysis caused by electrohydraulic cavitation [44]. HVED or other electrically assisted extractions are less thermally destructive than CE, and they are useful for extraction of specific thermolabile BACs. With increased effectiveness, such extracts are obtained at lower temperatures in a shorter period [45]. Another study found HVED as and efficient pre-treatment technique that intensifies pectin recovery from sugar beet pulp without modification of pectin structure and chemical composition [46]. For extraction of proteins and polyphenols from olive pit, HVED was found to be faster as compared to UAE or PEF [47]. This treatment retained more proteins and polyphenols in processing, thus demonstrating a promising technique for production of essential oils from oilseeds and herbs [48]. With generating electric fields and electrical discharges of up to 25 kV, there is additional formation of free radical species (ROS and RNS). This can be negative and destabilizing effect for sensitive bioactive compounds, especially in a long non-controlled treatment, where they can deteriorate [30]. This can be avoided by optimizing HVED treatment and shorter treatment time, as well to use gases in HVED treatments to reduce oxidation of phenolics and another compound of antioxidant potential.

**Figure 2:**
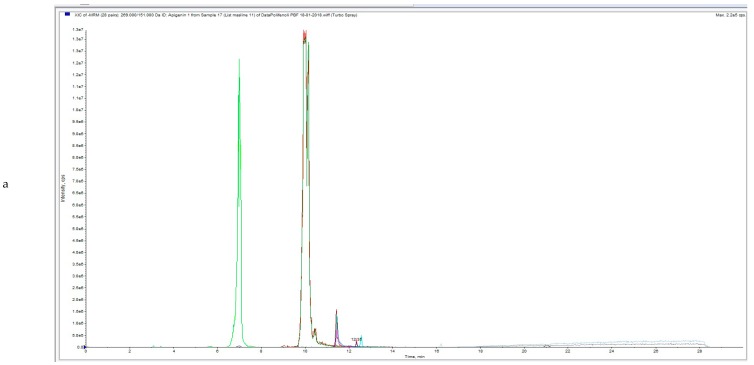

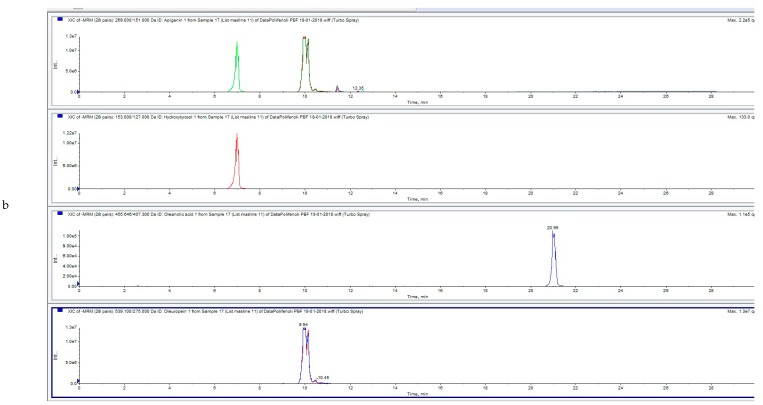
(**a**) UPLC-MS/MS analysis chromatogram of untreated OLE (3 OL50)—total ion current (TIC). (**b**) UPLC-MS/MS analysis chromatogram of untreated OLE (3 OL50).

**Figure 3:**
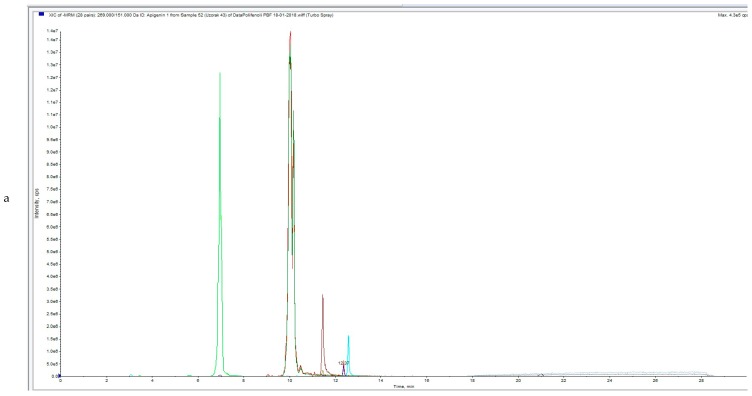

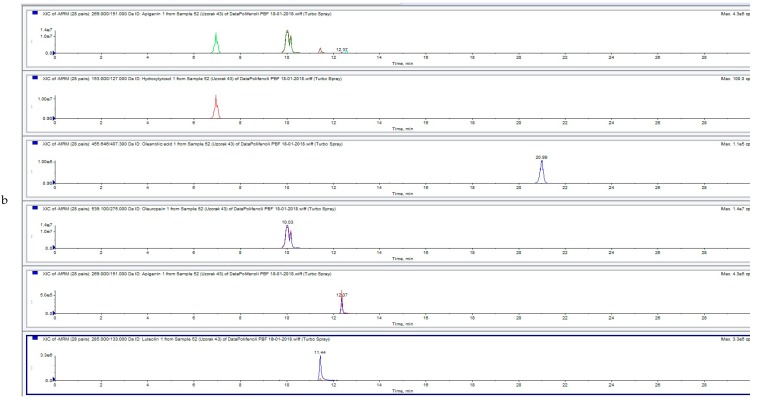
(**a**) UPLC-MS/MS analysis chromatogram of HVED treated (with nitrogen) OLE (OLN1)—total ion current (TIC). (**b**) UPLC-MS/MS analysis chromatogram of HVED treated (with nitrogen) OLE (OLN1).

**Figure 4:**
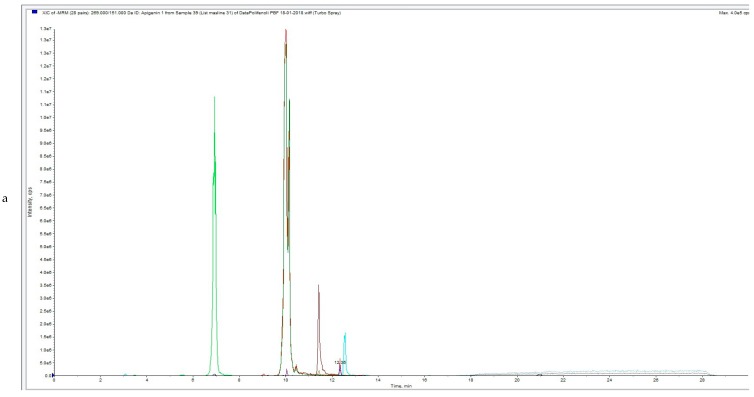

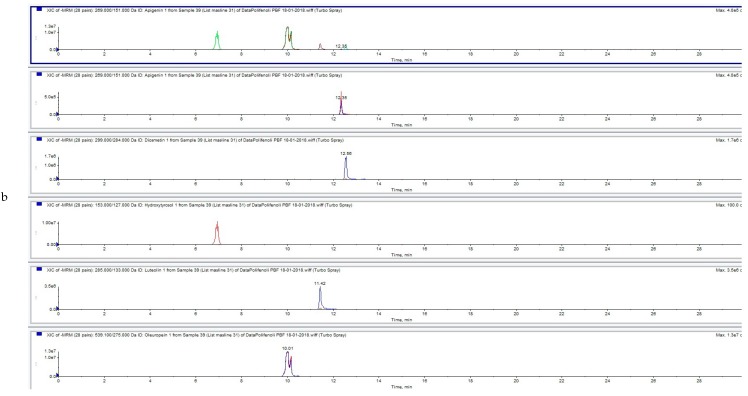
(**a**) UPLC-MS/MS analysis chromatogram of HVED treated (with argon) OLE (OLA1)—total ion current (TIC). (**b**) UPLC-MS/MS analysis chromatogram of HVED treated (with argon) OLE (OLA1).

UPLC-MS/MS analysis chromatograms of untreated and HVED treated OLs samples are shown in Figure 2a,b, Figure 3a,b, Figure 4a,b).

Figure 2a, Figure 3a and Figure 4a represent total ion chromatogram (TIC) of certain extract and in these chromatograms all MRM (multiple reaction monitoring) transitions of the method are included. On the x axis is intensity and on the y axis is time of elution or retention time (RT) of certain analyte.

Figure 2b, Figure 3b and Figure 4b represent extracted ion chromatogram (XIC) of certain extract and in these chromatograms only certain MRM-s transitions of the compounds are included. On the x axis is intensity and on the y axis is time of elution or retention time (RT) of certain analyte. In Figure 2b (Apigenin, Hydroxytyrosol, Oleanolic acid and Oleuropein), Figure 3b (Apigenin, Hydroxytyrosol, Oleanolic acid, Oleuropein and Luteolin) and Figure 4b Apigenin, Hydroxytyrosol, Diosmetin, Oleuropein and Luteolin).

### 3.4. Influence of HVED Treatment on Color Parameters of OLEs 

OLEs color parameters did not change significantly after the HVED treatments (Table 5). HVED treated OLEs had higher values of colorimetric variables a* (more red), b* (more yellowish), C (more tone color) and H (more saturated) than CE extracts, while average L* parameter was lower (darker samples) for HVED OLEs. Total difference in tone color (∆C), saturation (ΔH) and total color (ΔE) after HVED treatment was lower when using nitrogen gas comparing to argon. The highest difference in tone color (∆C) and total color difference (∆E) was observed for HVED samples with 50% ethanol (v/v) (OLN1, OLA1) and 9 min treatment (OLN6 and OLA5). The highest change in saturation (ΔH) was noted for HVED treated samples for 9 min (OLN7 and OLA5). Due to the electroporation effect of plant cells, there is a possibility of an extraction of different molecules depending on the diameter of the pore on electroporated plant membrane [29].

### 3.5. Influence of HVED Treatment and Electroporation Phenomena on Particle Size and Zeta Potential of OLEs

Immediately after electroporation, plant cells swell in four stages with leakage of ions to the outer cellular area. That is the period for the extraction of different molecules (including phenolics) that depends on the pore size. HVED treatment inflicts damage to the membranes by generating shock waves that are characteristic for the discharge. Hence, HVED has an important contribution to the mechanical damages, disintegration of cellular walls, homogenization and aggregation of cells. The results from electroporation on OLs are shown in Table 6. 

Physical properties of obtained extracts were tested for polydispersity index (PI) and zeta potential (mV). Data indicate possible usage of HVED technology in industry for simultaneous extraction and nanoformulation, i.e., for production of stable emulsion systems. From values of zeta potential, it is shown that the initial average diameter of particles in CE samples with ethanol are >300 nm. For HVED samples with argon or nitrogen and 25% ethanol content (*v*/*v*) (OLN 5, 6, 9, 12 and OLA5, 6, 9, 12) droplet diameter decreases, which is directly linked with negative zeta potential of −30 mV, indicating good stability against coalescence [49]. Zeta potential is often used as an indicator for droplet stability, where positive values above +30 mV and below negative −30 mV indicate good stability against coalescence are electrochemical balance. Therefore, by using OLs, in systems with 25% ethanol content (*v*/*v*) it is possible to produce stable emulsion systems and nanoparticles. 

The PI, which is a measure of homogeneity, was above 0.3. Ideally, PI should be <0.3, while values higher than 0.3 implicate broad distribution size and suggested particles are not monodispersed. Values for statistical analysis (Table 7 and Table 8) are indicating there was a statistically significant effect of ethanol content on ELS for argon samples, and for DLS for nitrogen. Also, it should be mentioned that HVED samples with water (OLA2, 3, 4, 10 and OLN2, 4, 10) had increased zeta potential and average diameter of particles, where surface charge of particles indicates formation of unstable particle systems.

### 3.6. Statistical Analysis of HVED Treatment Parameters and Ethanol Influence in Extraction of Bioactive Compound Form OLEs 

**Table 7:** Statistical significance for pH, conductivity, temperature difference, power, dynamic light scattering (DLS), polydispersity index (PI), electrophoretic light scattering (ELS), L*, a*, b*, total phenolic content (TPC), ferric reducing antioxidant power (FRAP), DPPH and yield of extraction. The MANOVA table decomposes the variability of each parameter into contributions due to various factors for OLE treated with argon.

Source		Main Effects			Interactions		
		A: Treatment Time	B: Voltage	C: Ethanol Content	AB	AC	BC
*p*-value	pH	0.1751	0.4805	0.0037	0.2276	0.5162	0.7081
	Conductivity	0.0431	0.5992	0.0017	0.2722	0.3039	0.8873
	Temperature difference	0.0867	0.0588	0.0564	0.1082	0.1968	0.6541
	Power	0.2380	0.0099	0.2097	0.3828	0.8125	0.2500
	DLS	0.6704	0.7696	0.3177	0.3569	0.6945	0.8880
	PI	0.4634	0.6328	0.2114	0.9126	0.7919	0.9259
	ELS	0.0703	0.1162	0.0163	0.2681	0.4337	0.1984
	L*	0.3500	0.5867	0.3631	0.5392	0.4922	0.6159
	a*	0.2793	0.6684	0.0474	0.5779	0.5319	0.7388
	b*	0.1225	0.8636	0.1252	0.8647	0.5679	0.3522
	TPC	0.1972	0.4714	0.1067	0.6437	0.6604	0.9387
	FRAP	0.6781	0.9773	0.1791	0.4250	0.8373	0.7394
	DPPH	0.7467	0.8024	0.2450	0.2872	0.6684	0.4903
	Yield of extraction	0.1982	0.4710	0.1072	0.6450	0.6633	0.9392

Where A determines treatment time, B stands for voltage and C stands for ethanol content. The *p*-values present the statistical significance of each of the factors.

**Table 8:** Statistical significance for pH, conductivity, temperature difference, power, DLS, PI, ELS, L*, a*, b*, TPC, FRAP, DPPH and yield of extraction. The MANOVA table decomposes the variability of each parameter into contributions due to various factors for OLs treated with nitrogen.

Source		Main Effects			Interactions		
		A: Treatment Time	B: Voltage	C: Ethanol Content	AB	AC	BC
*p* value	pH	0.1610	0.1516	0.0255	0.1209	0.2870	0.2384
	Conductivity	0.3678	0.7244	0.0128	0.4040	0.8518	0.9879
	Temperature Difference	0.1782	0.1677	0.0829	0.1081	0.2583	0.1489
	Power	0.5736	0.0046	0.4091	0.1835	0.1957	0.1552
	DLS	0.4132	0.3820	0.0277	0.3044	0.1810	0.0969
	PI	0.2785	0.4608	0.2353	0.7913	0.3141	0.5596
	ELS	0.2004	0.3594	0.0295	0.2676	0.1189	0.4675
	L*	0.6960	0.4382	0.2718	0.9382	0.6644	0.4497
	a*	0.4285	0.4968	0.0706	0.9572	0.5046	0.4027
	b*	0.4367	0.5694	0.2359	0.9952	0.4904	0.5976
	TPC	0.7034	0.8342	0.0652	0.7084	0.5300	0.5030
	FRAP	0.5573	0.2974	0.3888	0.6255	0.3050	0.6206
	DPPH	0.1798	0.8050	0.1974	0.1946	0.6913	0.4720
	Extraction Yield	0.7026	0.8341	0.0650	0.7089	0.5312	0.5062

Where A determines treatment time, B stands for voltage and C stands for ethanol content. The *p*-values present the statistical significance of each of the factors.

### 3.7. NIR and PCA Analysis of HVED Treated OLEs 

**Figure 5:**
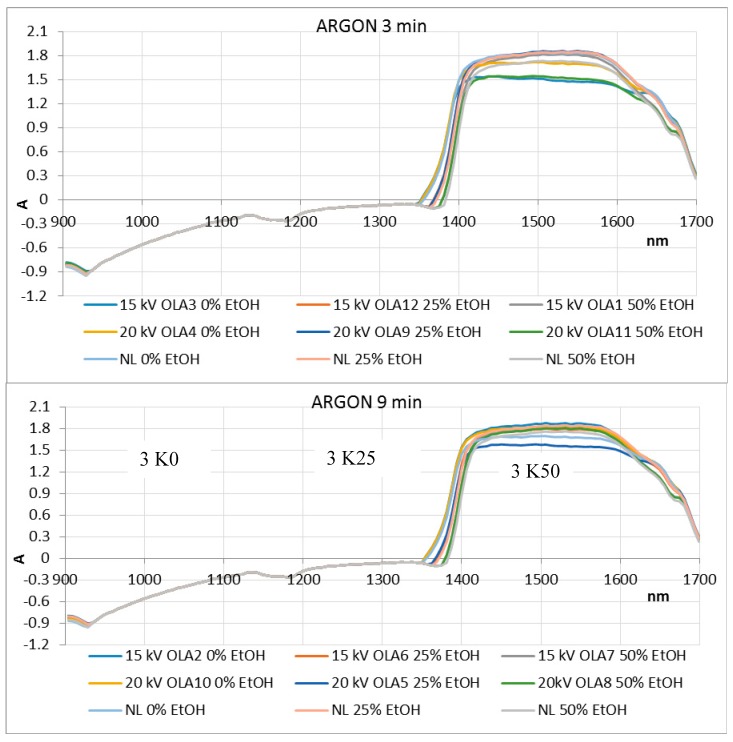
NIR spectra recorded in the wavelength range of 904 nm to 1699 nm for OLEs treated with argon for 3 and 9 min (NL: untreated olive leaves).

**Figure 6:**
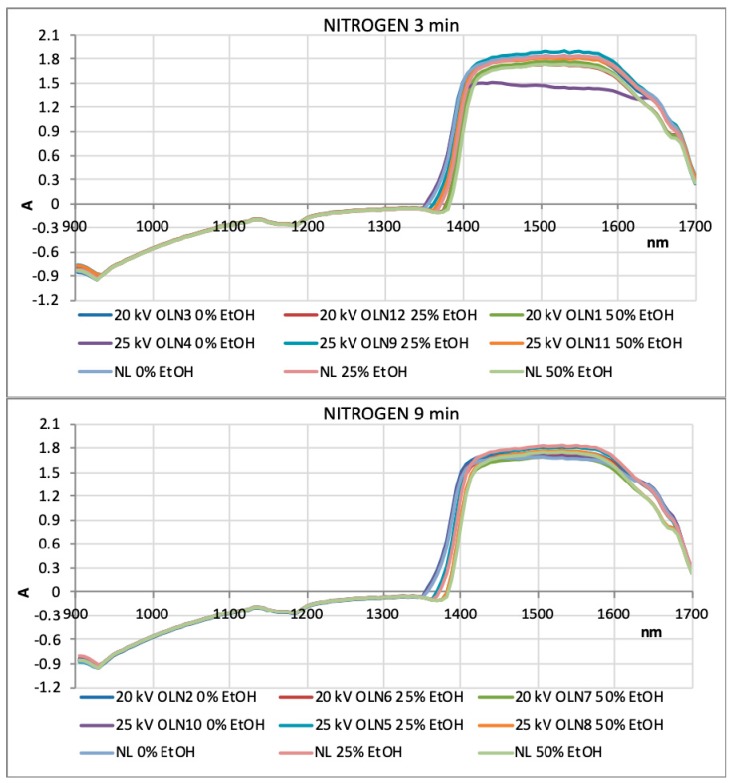
NIR spectra recorded in the wavelength range of 904 nm to 1699 nm for OLEs treated with nitrogen for 3 min and 9 min (NL: untreated olive leaves).

**Figure 7:**
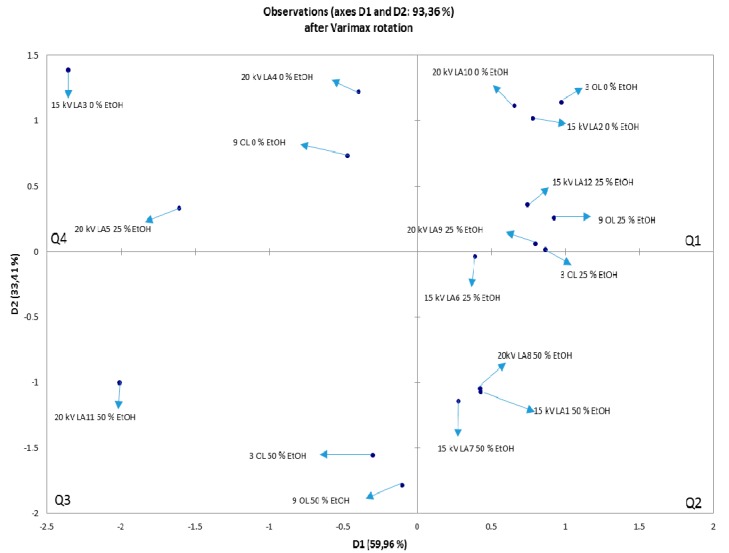
The PCA of untreated and HVED treated samples. The data is denoting untreated OLEs (CE), and OLEs treated with HVED using argon. Q stands for quadrant.

**Figure 8:**
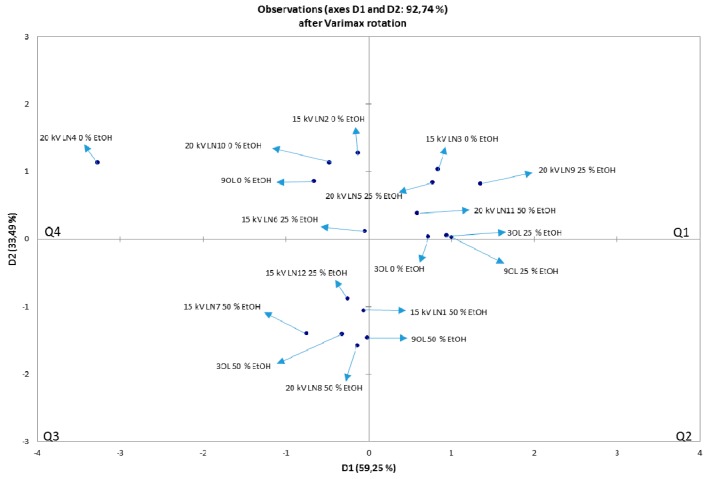
The PCA of untreated and HVED treated samples. The data is denoting untreated OLEs (CE), and OLEs treated with HVED using nitrogen. Q stands for quadrant.

NIR spectra were recorded in the wavelength range of 904–1699 nm for OLEs treated with argon and nitrogen for 3 and 9 min (Figure 5 and Figure 6). Based on their absorbance, they show no significant differences. However, in the ranges 904–928 and 1350–1699 nm, wavelength shifts are visible, indicating changes in the third and second overtone of the C–H and O–H relations. These relations are also associated with the hydroxyl group (–OH) bound directly to aromatic hydrocarbon group. The PCA was used for identifying patterns and highlighting similarities and differences in the data for an individual set of experiments. The goal of PCA was to extract the important information from the data table and express it as a set of new orthogonal variables, called principal components or factors. Figure 7 and Figure 8 show spatial projections defined by the first two main components labeled with F1 and F2 for HVED treated and untreated samples. Although several input variables described the variability of the whole system, often a large part of that variability is described by a small number of variables that are the main components. If this is met, the main components contain the same amount of information as input variables. In this case, the main components F1 and F2 comprise 93.36% variance of the original data for all the samples. Analysis of major components reveals connectivity among variables and allows interpretation that is otherwise difficult. The PCA analysis implies finding the values of the covariance matrix samples. The input data for the analysis of the main components are *p* variables and *n* observations (individual) and have a matrix shape *p* × *n*. The analysis begins with *p* variables data for *n* measurements (observations) using *k* main components (*k* < *p*) without losing system information. Main components represent the direction of maximum variability and provide a simpler description of the structure data set. The first principal component is the linear combination of the Y variables that accounts for the greatest possible variance, i.e., F1 (61.31%), while the other contains 32.05% variance. 

Figure 7 shows the distribution of samples with respect to the extraction parameters for argon treated and untreated samples, while Figure 8 shows the distribution for nitrogen treated and untreated samples. With higher proportion of ethanol in the solvent and with the longer treatment time, larger influence on the chemical compounds and origin of changes can be detected by NIR analysis (3th and 4th quadrant). For both PCA analysis, in the 1st and 2nd quadrant, there are samples extracted with pure water (0% ethanol) and 25% ethanol (*v*/*v*) or extracted for a shorter treatment time with or without HVED treatment. It can be seen the trend of motion patterns by the “strength” of the treatment in clockwise direction. Thus, in Figure 7, in 1st quadrant, samples were subdivided into 0 or 25% ethanol (*v*/*v*), treated for 3 or 9 minutes and a voltage of 15 or 20 kV. In the 4th quadrant there are samples extracted with 25 or 50% ethanol solution, treated for 3 or 9 minutes, 15 or 20 kV, while in 3rd quadrant there are samples that had powered treatment, meaning the extraction with 50% ethanol, untreated or HVED treated for 3 minutes at 20 kV. In Figure 8, in the 1st quadrant, samples were subdivided into 0 or 25% ethanol, treated for 3 minutes and with a voltage of 20 or 25 kV. In the 4th quadrant there are samples extracted with 25 or 50% ethanol, treated for 3 or 9 minutes, and 20 or 25 kV. In the 3rd quadrant is placed powered treated samples, meaning extraction with 25% or 50% ethanol, CE or HVED treatment for 3 or 9 minutes and voltages of 20 kV or 25 kV. The conclusion is that ethanol and applied HVED voltage have strong association with the extraction and corresponding changes in plant material. When ethanol is combined with HVED this destroys the linkage between phenolic compounds and components of the plant material to which they are bound.

### 3.8. Analysis of Pesticides and Metals in OL Samples

Table 9 and Table 10 show the content of pesticides and metals in initial samples taken for extraction. It is evident that samples have lower levels than set as maximum levels in REGULATION (EC) No 396/2005 on maximum residue levels (MRLs) of pesticides in or on food and feed of plant and animal origin and amending Council Directive 91/414/EEC and for metals according to COMMISSION REGULATION (EC) No 1881/2006. All extracts were compliant regarding content of contaminants, pesticide residues and toxic metals.

### 3.9. Critical Consideration on Costs of Equipment and Application of HVED for Extraction

The nonthermal processing market, was valued at USD 760.7 Million in 2016. It is projected to reach USD 1224.2 million by 2022, at a CAGR of 8.4% from 2017 reported by Markets and Markets™.

This aim of this paper and extensive analysis was to propose the possible application of HVED for the extraction of bioactive compounds from OLs. The aim was also, to emphasize use of water and ethanol as green solvents for efficient extraction of phenolic compounds. However, there should be critical discussion of the obtained results regarding costs of equipment and analysis, as well as short- and long-term improvements and possibilities of use of nonthermal technology like HVED. Regarding, nonthermal processing techniques like ultrasound, high pressure processing, pulsed electric fields, moderate electric fields, supercritical CO_2_ and subcritical water extraction, as extraction techniques, the high installation cost is a major restraining factor for this market. Inability to shift from conventional thermal technologies by large players is a major challenge for the nonthermal processing market. Therefore, there should be extensive research studies of environmental, economic and energy efficient nonthermal processing. 

The most used extraction techniques are the conventional techniques, like Soxhlet, heat reflux, boiling and distillation. These techniques are mostly based on the use of mild/high temperatures (50–90 °C) that can cause thermal degradation. The extraction efficiency is dependent of the correct solvent choice, and the agitation intensity in order to increase the solubility of materials and the mass transfer rate. CE is being reflected on long extraction times, high costs, usage of organic solvents and low extraction efficiency consequent low extraction yields. Also, there is potential for overheating of the matrix (herbs), high energy consumption and general cost.

The principal advantages of HVED against thermal conventional extraction procedures are related with the increased mass transfer, improved extraction yield, decreased processing time, decreased intensity of the conventional extraction parameters (i.e., extraction temperature, selection of solvents and solvent concentration), reduction of heat-sensitive compounds degradation (flavors etc.), facilitation of purified extract and reduction of energy costs and environmental impact.

For the highest energy inputs, in this study, there is a significant increase in conductivity, antioxidant activity and total phenolic compounds when compared with the control (a mixture of solvent in the same conditions as treated samples).

Therefore, a short-term overview is that HVED is an effective, but expensive technology, regarding the cost of equipment, high input cost due to the input feed gas (Ar, N_2_, He), and chemical residues and toxicological effects have not been studied yet. Possible application to scale-up of HVED to industry requires high capital cost and high energy consumption. Also, there are unresearched risks of corrosion rates of electrodes (the low-cost stainless steel has the highest corrosion rates).

However, long term potential is that there is low operational cost about electricity consumption (i.e., 90 kW/h × 0.05$/kW/h = 4.5 $/h). Advanced application of nonthermal techniques, like HVED, is that they can operate at room temperature ensuring that compound denaturation is avoided or at least decreased. HVED technology involves the application of short duration pulses (from several nanoseconds to several milliseconds) of high electric field strengths (20–80 kV cm^−1^) and relatively low energy (1–10 kJ/kg). HVED equipment comprises a high-voltage pulse generator, a treatment chamber with a suitable fluid handling system, system of gas delivery and a monitoring and controlling system. The herb (sample) is exposed to the electric field pulses in a static or continuous chamber with at least two electrodes, one on high voltage and the other at ground potential. The sample is submitted to a force per unit charge (the electric field) that is responsible for the cell disintegration (electroporation phenomena).

Therefore, there is long term sustainable application of electrotechnologies like HVED in pharma, food, cosmetics industry etc. 

## 4. Conclusions

The purpose of this extensive study was to evaluate and standardize advanced food technology, as HVED cold plasma-based tech, while accounting for green chemistry and modern sustainability and using autochthonous Mediterranean OLs (*Olea europaea* L.), so their extracts can be further available for industrial production. Such green extracts are abundant in antioxidants and phenolic components and are beneficial for human health and industrial production (food additives). 

HVED extraction resulted in effective extraction of phenolic components from leaves when comparing to CE. Highest values of phenolics in OLEs, were shown for HVED treatment with argon gas, 9 min treatment, 20 kV and 50% ethanol; and HVED treatment with nitrogen gas, 3 min, 20 kV and 50% ethanol. HVED extraction at the highest voltage (25 kV) and 9 min extraction time, resulted in the degradation of oleanolic acid in OLEs. This study confirmed that HVED is an extraction technique with significant yield due to disruptions of sample cells under electrical breakdown and enhanced mass transfer. For total phenolic content, all HVED samples had higher values (mg GAE/g) than untreated samples at the same temperature. The conclusion is that ethanol and applied HVED voltage, have strong linkage with changes caused by the treatment. Ethanol in combination with HVED, destroys the bond between phenolic compounds and plant material to which they are bound. From values of zeta potential, it is shown that the initial average diameter of particles in untreated samples with ethanol are around or below 300 nm. Hence, data indicate possible usage of HVED technology for simultaneous extraction and production of nanoemulsions. 

In conclusion, green OLEs obtained by green solvents (water/ethanol) and green technology with energy saving (HVED), matched the principles of green engineering and modern sustainability while having high nutritive value, e.g., they were rich in antioxidants and phenolic components [2,4,50].

## Figures and Tables

**Table 1:** Denotation of sample for high voltage electrical discharges (HVED) treatments.

ID	Sample	Treatment Time (min)	Voltage (kV)	Ethanol Content (%)	Stirring (min)	Extraction Type
1	3 OL0	0	0	0	3	CE
2	9 OL0	0	0	0	9	
3	3 OL25	0	0	25	3	
4	9 OL25	0	0	25	9	
5	3 OL50	0	0	50	3	
6	9 OL50	0	0	50	9	
7	OLN1	3	20	50	/	HVED
8	OLN2	9	20	0	/	
9	OLN3	3	20	0	/	
10	OLN4	3	25	0	/	
11	OLN5	9	25	25	/	
12	OLN6	9	20	25	/	
13	OLN7	9	20	50	/	
14	OLN8	9	25	50	/	
15	OLN9	3	25	25	/	
16	OLN10	9	25	0	/	
17	OLN11	3	25	50	/	
18	OLN12	3	20	25	/	
19	OLA1	3	15	50	/	
20	OLA2	9	15	0	/	
21	OLA3	3	15	0	/	
22	OLA4	3	20	0	/	
23	OLA5	9	20	25	/	
24	OLA6	9	15	25	/	
25	OLA7	9	15	50	/	
26	OLA8	9	20	50	/	
27	OLA9	3	20	25	/	
28	OLA10	9	20	0	/	
29	OLA11	3	20	50	/	
30	OLA12	3	15	25	/	

OL = olive leaf, N = nitrogen, A = argon. For HVED, numbers 1–12 are the order of conducted treatment. For CE treatments, 3 and 9 are referred to treatment time (min) while 0, 25, and 50 stands for concentration of an ethanol solvent (%).

**Table 2:** Average values of pH, conductivity (μS/cm) for untreated and HVED treated samples, starting temperature (°C), final temperature (°C) and power (kW) after HVED treatments.

ID	Sample	pH	Conductivity (μS/cm)	Starting Temperature (°C)	Final Temperature (°C)	Power (kW)	Extraction Type
1	3 OL0	5.54 ± 0.21	516.0 ± 4	20.8 ± 0.9	20.8 ± 0.5	/	CE
2	9 OL0	5.45 ± 0.11	349.0 ± 3	19.7 ± 0.4	19.7 ± 0.4	/	
3	3 OL25	6.07 ± 0.09	139.2 ± 2.3	20.1 ± 0.2	20.1 ± 0.6	/	
4	9 OL25	6.21 ± 0.15	141.6 ± 3.2	20.0 ± 0.5	20.0 ± 0.6	/	
5	3 OL50	6.88 ± 0.12	36.6 ± 1.1	21.0 ± 0.4	21.0 ± 0.4	/	
6	9 OL50	6.51 ± 0.11	41.3 ± 1.2	21.0 ± 0.7	21.0 ± 0.2	/	
7	OLN1	6.46 ± 0.22	51.4 ± 1.2	26.4 ± 0.8	26.7 ± 0.8	12 ± 1	HVED
8	OLN2	5.80 ± 0.16	242.4 ± 3.3	24.2 ± 0.6	24.9 ± 0.3	12 ± 1	
9	OLN3	5.87 ± 0.14	272.1 ± 2.3	25.2 ± 0.5	25.6 ± 0.6	12 ± 1	
10	OLN4	5.65 ± 0.21	224.3 ± 3.2	24.8 ± 0.6	26.2 ± 0.6	19 ± 1	
11	OLN5	5.93 ± 0.19	124.7 ± 1.2	23.4 ± 0.3	23.6 ± 0.5	17 ± 0	
12	OLN6	5.85 ± 0.18	126.5 ± 2.5	24.3 ± 0.4	23.8 ± 0.4	12 ± 1	
13	OLN7	6.11 ± 0.17	63.4 ± 1.3	23.5 ± 0.5	23.3 ± 0.6	10 ± 1	
14	OLN8	6.09 ± 0.21	57.9 ± 1.1	22.7 ± 0.7	23.3 ± 0.3	20 ± 1	
15	OLN9	6.00 ± 0.15	93.4 ± 1.2	25.5 ± 0.3	25.7 ± 0.4	19 ± 1	
16	OLN10	5.78 ± 0.11	275.6 ± 3.1	23.3 ± 0.2	28.4 ± 0.6	22 ± 1	
17	OLN11	6.10 ± 0.12	51.9 ± 1.1	28.2 ± 0.5	26.7 ± 0.7	19 ± 0	
18	OLN12	5.99 ± 0.17	104.6 ± 1.2	25.9 ± 0.4	26.1 ± 0.6	14 ± 1	
19	OLA1	6.45 ± 0.16	53.2 ± 1.1	23.7 ± 0.6	23.8 ± 0.6	7 ± 1	
20	OLA2	5.30 ± 0.15	268.3 ± 3.4	23.6 ± 0.7	25.6 ± 0.6	9 ± 1	
21	OLA3	5.21 ± 0.12	250.6 ± 4.5	23.6 ± 0.7	24.7 ± 0.5	10 ± 1	
22	OLA4	5.28 ± 0.19	233.3 ± 3.2	23.7 ± 0.5	25.1 ± 0.4	14 ± 1	
23	OLA5	5.77 ± 0.12	126.4 ± 2.3	24.3 ± 0.3	26.1 ± 0.3	16 ± 0	
24	OLA6	5.88 ± 0.11	127.5 ± 2.3	24.0 ± 0.4	24.2 ± 0.6	7 ± 0	
25	OLA7	6.32 ± 0.12	61.9 ± 1.6	23.9 ± 0.6	23.1 ± 0.6	9 ± 1	
26	OLA8	6.25 ± 0.13	59.0 ± 1	23.2 ± 0.4	24.4 ± 0.5	13 ± 1	
27	OLA9	6.00 ± 0.15	94.6 ± 2.0	23.8 ± 0.6	24.5 ± 0.6	14 ± 1	
28	OLA10	5.18 ± 0.16	285.7 ± 3.8	24.5 ± 0.7	29.9 ± 0.4	16 ± 1	
29	OLA11	6.36 ± 0.17	49.6 ± 1.3	24.5 ± 0.6	24.6 ± 0.7	12 ± 0	
30	OLA12	5.88 ± 0.19	105.9 ± 2.8	24.2 ± 0.5	24.0 ± 0.6	7 ± 1	

OL = olive leaf, N = nitrogen, A = argon. For HVED, numbers 1–12 are the order of conducted treatment. For CE treatments, 3 and 9 are referred to treatment time (min) while 0, 25, and 50 stands for concentration of an ethanol solvent (%).

**Table 3:** Concentrations of total phenolic content (TPC) values, antioxidant capacity (AOC) (2,2-Diphenyl-2-Picrylhydrazyl - DPPH) and ferric reducing antioxidant power (FRAP)) and extraction yield in extracts produced by conventional extraction (CE) and HVED.

ID	Sample	TPC (mg GAE/g)	DPPH (µmol TAE/g)	FRAP (µmol FE/g)	Yield (%)	Extraction Type
1	3 OL0	5.32 ± 0.02	27.06 ± 0.01	63.36 ± 2.24	0.53 ± 0.00	CE
2	9 OL0	15.85 ± 0.11	31.10 ± 0.08	146.93 ± 3.54	1.58 ± 0.01	
3	3 OL25	13.35 ± 0.08	24.78 ± 0.05	150.50 ± 4.57	1.33 ± 0.01	
4	9 OL25	16.04 ± 0.10	30.46 ± 0.07	179.07 ± 6.09	1.60 ± 0.01	
5	3 OL50	14.06 ± 0.09	32.71 ± 0.06	159.79 ± 5.42	1.41 ± 0.01	
6	9 OL50	20.61 ± 0.14	33.31 ± 0.08	221.93 ± 6.99	2.06 ± 0.01	
7	OLN1	49.21 ± 0.10	31.49 ± 0.07	266.21 ± 7.53	4.92 ± 0.01	HVED
8	OLN2	29.82 ± 0.07	28.60 ± 0.05	208.36 ± 4.28	2.98 ± 0.01	
9	OLN3	18.08 ± 0.08	25.96 ± 0.06	197.64 ± 2.00	1.81 ± 0.01	
10	OLN4	33.12 ± 0.12	27.46 ± 0.08	199.79 ± 5.22	3.31 ± 0.01	
11	OLN5	24.17 ± 0.05	31.31 ± 0.04	374.79 ± 6.74	2.42 ± 0.01	
12	OLN6	26.95 ± 0.08	30.14 ± 0.09	308.36 ± 4.45	2.69 ± 0.01	
13	OLN7	45.47 ± 0.06	30.49 ± 0.10	209.79 ± 2.01	4.55 ± 0.01	
14	OLN8	47.21 ± 0.09	31.81 ± 0.08	229.79 ± 5.82	4.72 ± 0.01	
15	OLN9	29.99 ± 0.07	28.35 ± 0.01	301.21 ± 4.68	3.00 ± 0.01	
16	OLN10	28.95 ± 0.11	29.99 ± 0.08	256.21 ± 5.74	2.89 ± 0.01	
17	OLN11	45.82 ± 0.16	27.96 ± 0.07	561.93 ± 9.11	4.58 ± 0.02	
18	OLN12	35.03 ± 0.10	31.53 ± 0.02	284.07 ± 3.21	3.50 ± 0.01	
19	OLA1	39.56 ± 0.08	30.78 ± 0.15	234.07 ± 2.66	3.96 ± 0.01	
20	OLA2	32.69 ± 0.05	26.53 ± 0.08	145.50 ± 2.87	3.27 ± 0.00	
21	OLA3	9.82 ± 0.04	29.71 ± 0.11	97.64 ± 1.15	0.98 ± 0.00	
22	OLA4	26.69 ± 0.02	25.53 ± 0.08	169.07 ± 5.92	2.67 ± 0.00	
23	OLA5	36.17 ± 0.04	29.60 ± 0.17	315.50 ± 4.00	3.62 ± 0.00	
24	OLA6	31.21 ± 0.09	30.64 ± 0.02	321.21 ± 6.38	3.12 ± 0.01	
25	OLA7	53.64 ± 0.14	29.85 ± 0.12	443.36 ± 5.21	5.36 ± 0.01	
26	OLA8	65.99 ± 0.06	31.53 ± 0.01	237.64 ± 3.73	6.60 ± 0.01	
27	OLA9	30.77 ± 0.05	26.81 ± 0.08	326.21 ± 2.16	3.08 ± 0.01	
28	OLA10	21.21 ± 0.04	29.67 ± 0.05	196.21 ± 2.97	2.12 ± 0.00	
29	OLA11	42.60 ± 0.11	32.53 ± 0.10	343.36 ± 3.01	4.26 ± 0.01	
30	OLA12	26.43 ± 0.01	30.03 ± 0.08	354.07 ± 5.36	2.64 ± 0.00	

OL = olive leaf, N = nitrogen, A = argon. For HVED, numbers 1–12 are the order of conducted treatment. For CE treatments, 3 and 9 are referred to treatment time while 0, 25, and 50 stands for concentration of an ethanol solvent (%). Percentage of yield was calculated as (g GAE/g of sample) × 100.

**Table 4:** Concentration of individual phenolic compounds in CE and HVED olive leaves extracts (OLEs) (ng/mL).

ID	Sample	Apigenin	Diosmetin	Hydroxytyrosol	Luteolin	Oleanolic Acid	Oleuropein	Quercentin	Extraction Type
1	3 OL0	156	124	9.17	2356.6	4.45	50.4	N/A	CE
2	9 OL0	172	121	11.6	71.5	2.12	56.2	0.054	
3	3 OL25	125	112	494	550	0.66	3959	0.140	
4	9 OL25	144	132	125	580	0.79	825	0.124	
5	3 OL50	31.9	14.5	4120	154	3918	23485	247	
6	9 OL50	193	154	13.0	80.8	8.37	108	3.01	
7	OLN1	58.6	47.0	3016	353	3386	17646	9.07	HVED
8	OLN2	157	118	86.6	138	8.84	99.3	N/A	
9	OLN3	109	101	97.5	96.3	3.65	76.1	0.048	
10	OLN4	127	98.1	96.5	122	N/A	74.2	0.042	
11	OLN5	116	119	80.4	361	1.83	163	0.109	
12	OLN6	134	127	67.5	424	2.92	165	0.115	
13	OLN7	38.9	30.0	3090	255	2803	17311	7.17	
14	OLN8	44.3	33.0	3294	265	2705	18413	9.0	
15	OLN9	149	159	108	375	8.57	168	0.179	
16	OLN10	111	94.2	192	252	3.76	178	0.103	
17	OLN11	106	106	97.5	403	2.00	424	0.121	
18	OLN12	56.8	49.2	3426	386	2851	17388	8.54	
19	OLA1	53.6	59.9	2964	357	2004	16103	5.27	
20	OLA2	198	76.6	10.1	29.8	5.74	57.4	0.043	
21	OLA3	124	130	112	215	2.41	97.9	0.032	
22	OLA4	113	100	64.0	113	N/A	52.5	0.021	
23	OLA5	110	123	128	487	N/A	247	0.146	
24	OLA6	115	122	145	457	N/A	315	0.218	
25	OLA7	60.1	60.6	3660	431	1844	17127	6.43	
26	OLA8	48.2	41.5	3682	334	2043	17895	15.6	
27	OLA9	111	116	149	480	6.22	465	0.189	
28	OLA10	94.2	85.3	139	166	N/A	102	0.061	
29	OLA11	51.4	51.0	2967	372	2193	16695	5.79	
30	OLA12	142	141	77.2	459	7.51	166	0.191	

OL = olive leaf, N = nitrogen, A = argon. For HVED, numbers 1–12 are the order of conducted treatment. For CE treatments, 3 and 9 are referred to treatment time (min) while 0, 25, and 50 stands for concentration of an ethanol solvent (%).

**Table 5:** CIELab color parameters of CE and HVED treated OLEs.

ID	Sample	L*	a*	b*	C	H	∆C	∆H	∆E	Extraction Type
1	3 OL0	81.86	4.96	42.70	42.99	1.46	/	/	/	CE
2	9 OL0	82.06	6.59	46.76	47.22	1.43	/	/	/	
3	3 OL25	89.56	0.06	30.53	30.53	1.57	/	/	/	
4	9 OL25	91.02	−0.37	31.26	31.26	−1.56	/	/	/	
5	3 OL50	94.12	−3.82	27.89	28.15	−1.43	/	/	/	
6	9 OL50	91.03	−3.82	38.68	38.87	−1.47	/	/	/	
7	OLN1	80.34	−2.69	45.25	45.33	−1.51	17.18	2.74	22.19	HVED
8	OLN2	79.75	7.78	44.90	45.57	1.40	−1.65	1.46	3.20	
9	OLN3	81.28	6.06	42.60	43.03	1.43	0.04	1.10	1.25	
10	OLN4	80.38	4.01	40.60	40.80	1.47	−2.19	0.72	2.74	
11	OLN5	82.35	1.17	29.06	29.08	1.53	−2.18	1.57	9.08	
12	OLN6	81.25	−2.59	45.68	45.75	−1.51	14.49	1.69	17.56	
13	OLN7	84.44	2.87	36.30	36.41	1.49	−2.45	6.66	9.69	
14	OLN8	86.03	−3.34	42.78	42.91	−1.49	4.04	0.84	6.48	
15	OLN9	83.26	0.59	26.29	26.30	1.55	−4.23	0.58	7.61	
16	OLN10	80.90	6.73	47.19	47.67	1.43	0.45	0.08	1.25	
17	OLN11	88.01	−3.70	40.73	40.90	−1.48	12.75	1.54	14.22	
18	OLN12	85.42	0.55	27.78	27.79	1.55	−2.74	0.52	4.99	
19	OLA1	84.82	−2.01	41.69	41.74	−1.52	13.59	3.01	16.74	
20	OLA2	78.28	10.44	52.28	53.31	1.37	6.09	2.86	7.72	
21	OLA3	86.90	3.20	41.42	41.54	1.49	−1.44	1.63	5.49	
22	OLA4	80.40	8.10	48.30	48.97	1.40	5.99	2.32	6.58	
23	OLA5	80.45	4.78	47.39	47.63	1.47	16.37	4.33	19.96	
24	OLA6	83.55	2.67	41.24	41.33	1.51	10.06	2.75	12.83	
25	OLA7	83.12	−1.37	43.09	43.11	−1.54	4.24	2.73	9.38	
26	OLA8	85.95	−2.71	38.36	38.46	−1.50	−0.41	1.08	5.21	
27	OLA9	82.62	2.21	37.57	37.63	1.51	7.10	1.92	10.12	
28	OLA10	78.98	9.06	50.87	51.67	1.39	4.45	1.79	5.70	
29	OLA11	84.57	−2.25	36.37	36.44	−1.51	8.29	2.38	12.87	
30	OLA12	82.27	2.50	36.80	36.88	1.50	6.35	2.21	9.92	

OL = olive leaf, N = nitrogen, A = argon. For HVED, numbers 1–12 are the order of conducted treatment. For CE treatments, 3 and 9 are referred to as treatment time while 0, 25, and 50 stands for concentration of an ethanol solvent (%). L*—lightness from black to white; a* from green to red, and b* from blue to yellow; C—chroma/tone color; H—hue angle; ∆C—total tone color difference; ΔH—total saturation difference; ΔE—total color difference.

**Table 6:** Results of particle size and zeta potential analysis for CE and HVED treated samples.

ID	Sample	Z Average Mean (d·nm)	PI	Zeta potential (mV)	Extraction Type
1	3 OL0	541.55 ± 6.38	0.62 ± 0.01	−9.31 ± 0.47	CE
2	9 OL0	370.60 ± 4.57	0.69 ± 0.06	−7.96 ± 0.25	
3	3 OL25	270.45 ± 3.65	0.63 ± 0.11	−11.30 ± 2.44	
4	9 OL25	303.25 ± 4.56	0.68 ± 0.08	−8.98 ± 1.12	
5	3 OL50	333.10 ± 4.58	0.54 ± 0.00	−10.43 ± 0.81	
6	9 OL50	166.50 ± 2.92	0.56 ± 0.06	−18.07 ± 0.12	
7	OLN1	394.60 ± 3.81	0.41 ± 0.03	−27.13 ± 1.46	HVED
8	OLN2	672.00 ± 7.83	0.48 ± 0.06	−15.77 ± 0.30	
9	OLN3	751.35 ± 9.64	0.73 ± 0.04	−17.79 ± 0.66	
10	OLN4	1545.50 ± 15.01	0.65 ± 0.04	−18.87 ± 1.45	
11	OLN5	139.30 ± 2.45	0.32 ± 0.04	−53.77 ± 0.27	
12	OLN6	203.70 ± 3.65	0.53 ± 0.05	−45.99 ± 5.52	
13	OLN7	653.15 ± 6.83	0.95 ± 0.14	−22.21 ± 0.37	
14	OLN8	378.15 ± 3.63	0.59 ± 0.07	−33.25 ± 1.46	
15	OLN9	279.15 ± 3.72	0.28 ± 0.01	−28.09 ± 0.88	
16	OLN10	952.40 ± 9.43	0.71 ± 0.10	−16.43 ± 0.10	
17	OLN11	193.50 ± 1.23	0.36 ± 0.08	−31.94 ± 2.48	
18	OLN12	304.05 ± 4.72	0.32 ± 0.04	−36.44 ± 2.44	
19	OLA1	452.90 ± 6.74	0.60 ± 0.06	−23.81 ± 0.75	
20	OLA2	1551.50 ± 16.63	0.85 ± 0.13	−19.76 ± 0.37	
21	OLA3	318.20 ± 3.74	0.42 ± 0.05	−16.96 ± 0.40	
22	OLA4	846.95 ± 8.72	0.71 ± 0.15	−17.53 ± 0.23	
23	OLA5	177.25 ± 1.53	0.43 ± 0.04	−54.14 ± 2.72	
24	OLA6	183.25 ± 1.74	0.26 ± 0.00	−36.95 ± 0.26	
25	OLA7	447.40 ± 2.82	0.50 ± 0.11	−29.91 ± 0.03	
26	OLA8	369.40 ± 2.76	0.63 ± 0.06	−35.07 ± 0.73	
27	OLA9	198.45 ± 1.34	0.31 ± 0.03	−41.08 ± 4.01	
28	OLA10	506.95 ± 5.45	0.69 ± 0.06	−19.41 ± 0.10	
29	OLA11	522.55 ± 6.28	0.48 ± 0.10	−23.29 ± 1.53	
30	OLA12	164.70 ± 1.28	0.46 ± 0.00	−35.08 ± 5.12	

OL = olive leaf, N = nitrogen, A = argon. For HVED, numbers 1–12 are the order of conducted treatment. For CE treatments, 3 and 9 are referred to treatment time (min) while 0, 25, and 50 stands for concentration of an ethanol solvent (%).

**Table 9:** Residue levels and maximum residue levels (MRLs) of pesticides (mg/kg) in olive leaves (OLs) samples.

Pesticides	MRL mg/kg	Content mg/kg
Alachlor	0.02	<0.005
Aldrin and Dieldrin (Aldrin and dieldrin combined expressed as dieldrin)	0.01	<0.002
Captan (Sum of captan and THPI, expressed as captan)	0.06	<0.020
DDT (sum of p,p´-DDT, o,p´-DDT, p-p´-DDE and p,p´-TDE (DDD) expressed as DDT)	0.05	<0.004
Endosulphan (sum of alpha- and beta-isomers and endosulphan-sulphate expresses as endosulphan)	0.05	<0.002
Endrin	0.01	<0.004
Heptachlor (sum of heptachlor and heptachlor epoxide expressed as heptachlor)	0.01	<0.002
Hexachlorobenzene	0.01	<0.002
Hexachlorocyclohexane (HCH), alpha-isomer	0.01	<0.002
Hexachlorocyclohexane (HCH), beta-isomer	0.01	<0.002
Iprodione	20	<0.010
Lindane (Gamma-isomer of hexachlorocyclohexane (HCH))	0.01	<0.002
Methoxychlor	0.01	<0.010
Tolylphluanid (Sum of tolylphluanid and dimethylaminosulphotoluidide expressed as tolylphluanid)	0.05	<0.020
Vinclozolin	0.02	<0.002

**Table 10:** Residue levels and MRLs of metals.

Metals	MRL mg/kg	Content mg/kg
Lead (Pb)	3.00	<0.050
Cadmium (Cd)	1.00	0.011
Mercury (Hg)	0.50	0.023
Arsenic (As)	-	<0.005
Chromium (Cr)	-	0.240
Nickel (Ni)	-	1.82
Manganese (Mn)	-	52.0
Iron (Fe)	-	101
Copper (Cu)	-	9.70
Zinc (Zn)	-	15.0
